# Influence of Friction Riveting Parameters on the Dissimilar Joint Formation and Strength

**DOI:** 10.3390/ma15196812

**Published:** 2022-09-30

**Authors:** Damjan Klobčar, Franci Pušavec, Drago Bračun, Ivica Garašić, Zoran Kožuh, Aleksandar Vencl, Uroš Trdan

**Affiliations:** 1 Faculty of Mechanical Engineering, University of Ljubljana, Aškerčeva 6, 1000 Ljubljana, Slovenia; 2 Faculty of Mechanical Engineering and Naval Architecture, University of Zagreb, 10000 Zagreb, Croatia; 3 University of Belgrade, Faculty of Mechanical Engineering, 11120 Belgrade, Serbia; 4 South Ural State University, 454080 Chelyabinsk, Russia

**Keywords:** friction riveting, 2024-T351 aluminium alloy, PEI polymer, X-ray imaging, pull-out force

## Abstract

Friction riveting represents a promising technology for joining similar and/or dissimilar materials of light-weight components. However, the main drawback of the technology is that it is primarily used only with special machines for friction welding that have a force control. In this study we used accessible CNC machines with a position control. A set of friction riveting experiments was performed to establish the relationship between the processing parameters, the rivet formation and its mechanical strength. During the manufacturing process, the axial force and torque were constantly measured. The fabricated joints were examined using an X-ray imaging technique, microstructural analyses, and mechanical tests. The samples were subjected to the pull-out test to analyse the joints’ strength and determine the failure mode type. In addition, a correlation between the friction riveting processing parameters, the rivet penetration depth, the rivet shape and the joint strength was established. The results depict that a higher axial force in the first production phase at the higher feeding rate increases the penetration depth, while in the second phase at lower feeding rate, an anchoring shape of a rivet forms.

## 1. Introduction

In the light-weight design of the components that are used for aerospace applications, in wind power plants and in the automotive industry, for instance, an optimal combination of different materials is the key requirement to obtain the desired microstructural and mechanical properties, and thereby improve the functionality of the part [1,2,3]. The materials are selected based on their unique properties (mechanical, electrical, physical and chemical) and their strength-to-weight ratio. Different materials are used such as advanced high strength steels (AHSS), magnesium alloys, aluminium alloys, titanium alloys, nickel based super alloys, stainless steels, fibre reinforced composites, etc. [4,5]. An important issue in the production of such multi-material components is in their manufacturing (forming, milling and lamination) and their joining [6,7]. The joining of similar materials could present a challenge regarding the reliability of the joint, its safety and the possibilities for their certification. At the end of the product’s lifetime, an important issue is its recyclability and ability to be reused to make the value chain more sustainable. When dissimilar materials need to be joined together and the challenges of this are even more demanding [8,9]. For the adhesive bonding of dissimilar materials, rivets or bolts are frequently used in a hybrid joint in order to enhance the joint safety and to obtain the required certification [10]. The following hybrid joining techniques can be used for this purpose: (*i*) a combination of adhesive bonding with mechanical joining [11] (e.g., hem flange bonding, adhesive bonding in conjunction with mechanical point joints, adhesive bonding and clinching [12,13], adhesive bonding and self-piercing or riveting, adhesive bonding and blind riveting, adhesive bonding combined with other mechanical fasteners), (*ii*) adhesive bonding combined with solid state joining techniques, (*iii*) adhesive injection fasteners, (*iv*) a combination of adhesive bonding with fusion welding (e.g., adhesive bonding combined with resistance spot welding [14], adhesive bonding combined with other fusion welding processes), (*v*) combinations of fusion welding techniques (e.g., Laser-arc welding processes, Laser-MIG welding [15], Laser-TIG welding, Laser-Plasma arc welding, laser-MIG tandem welding process, MIG Plasma welding, etc.), and (*vi*) friction self-piercing riveting [16,17,18]; in which a combination of two mechanical joining techniques is applied [19,20,21]. However, the last-mentioned technique, the lags well behind the other ones and it is, therefore, worth investigating.

Friction riveting has been developed as an alternative technology to riveting and bolting for joining polymer–metal multimaterials [22,23]. In these studies, the aluminium threaded rivets were used, and the joining mechanisms, microstructures, tensile and shear strengths and temperature evolutions were investigated. The process temperatures reached about 500 °C, which is 95% of the melting point of the aluminium rivet and corresponds to the temperature of thermal degradation of the PEI polymer that was used. The tensile strength achieved 95% of the rivet strength and 70% of the shear strength of the polymer that was used.

Further studies were conducted on the friction riveting of grade 2 titanium threaded rivets and glass fibre-reinforced polyetherimide (GFRP) [24,25]. In the first study, a volumetric ratio was introduced to determine the anchoring effect and correlate it with the tensile strength. However, a moderate tensile strength was obtained with the rivet pull-out test [24]. A later study focused on the feasibility of using friction rivets to connect emergency bridges [25]. It was found that the shear strength of the overlap reached up to 200 MPa, which is similar to the mechanical strength of the bolted connections. Proenca et al. [26] investigated the friction riveting of aluminium alloy 6058-T6 and GFR polyamide 6. The study was conducted with a force control, aiming to produce a rivet with a suitable depth and an appropriate anchoring shape. A two-stage friction riveting process was carried out, and the tensile strength of the joint reached 95% of the metal rivet.

Proenca et al. [27] found the influence that rotation speed had on the anchorage of the rivet and the mechanical strength. Cipriano et al. [28,29] studied the process of force-controlled friction riveting on the joint formation and its mechanical strength and energy efficiency. A detailed statistical study was conducted in which statistical models were developed to predict the anchorage shape and depth as well as the strength as a function of the processing parameters. Borba et al. [30] investigated the mechanical properties of friction-riveted joints in composite laminates under quasi-static and cyclic loading. They found that the plastically deformed rivet acts as a crack arrestor, which extends the fatigue life of the friction-riveted joints in comparison to that of the bolted joints.

In another study, Cipriano et al. [31] investigated the single-phase friction riveting of a PEI polymer using rivets that were made from a 5 mm thick AA2024-T351 alloy, omitting the forging phase. Extensive experiments and analyses were carried out to prove the feasibility of the process and to achieve comparable mechanical properties and the energy efficiency to the two-phase process. Mallmann et al. [32] investigated the single-phase friction riveting of AA6056-T6 rivets with 3D-printed carbon fibre-reinforced polyamide 6 with different carbon fibre contents. The strength of the joint reached more than 90% of the strength of the base material, with a failure being caused by the aluminium rivet losing some of its mechanical properties due to the temperature rise. Feier et al. [33] investigated the low-cost friction riveting method using a hand drill and a rivet that was made of AA2024-T351 alloy and a PEI polymer. The results confirmed the occurrence of an undesired failure in the rivet pull-out test, which was analysed in detail using FE modelling. Cipriano et al. [34] continued the research with the thermomechanical modelling of the friction riveting process. They analysed, in detail, the plastic deformation of the rivet, the temperature distribution and the heat input to simulate the process and provide a better understanding of it. In addition, Sankaranarayanan et al. [35] reviewed several research papers on friction riveting for joining lightweight multi-material structures and compared the advantages and disadvantages of the process.

Hence, the current study was focused on the friction riveting of an aluminium rivet to a polymer base. However, since this process usually demands the use of a special friction welding machine with a force control tool, research facilities do not allow the general use of the process. In view of this, a standard computer numeric control (CNC) milling machine with a position control tool has been used for the friction riveting process. Since such machines are versatile and widely used; they can be easily accessed and used. The aim of research was to accomplish the same or better joint strength as has already been accomplished, by developing a position control protocol using more accessible, low-cost CNC machines. The produced joints were subjected to visual observations, X-Ray examination, microscopy, and mechanical testing (pull-out test) in order to establish a precise correlation between the process parameters, the final rivet shape, the anchoring rivet depths and the overall joint strength. 

## 2. Materials and Methods

Based on an extensive literature survey of the most recent literature in the field of friction riveting, the detailed planning of the experiments (Table 1) was conceived. The chosen material for the rivets was aluminium alloy (AA) 2024-T351, of which the chemical composition, mechanical and physical properties are shown in Table 2 and Table 3, respectively. From this, material samples of 6 mm in diameter with a 60 mm length were prepared. The second material, which was being used to rivet it, was polyetherimide (PEI) (Table 4), from which sample blocks of 24 × 24 × 14 mm^3^ were prepared. Friction riveting experiments were performed using the 5-axis CNC machining centre Doosan NX 6500 II (DN Solutions, Jeongdong, Korea). During the experiments, the measuring protocol was prepared in which the processing axial force and torque were constantly measured using a Kistler piezoelectric dynamometer, Kistler charge amplifier, a measuring card (NI 9215) and a DAQExpress, both from National instruments (Austin, TX, USA).

All of the experiments were performed using a constant rotation speed of 19,000 rpm. In the initial phase, the rivet feeding speed was set to 10 mm/min, until a depth of −0.15 mm was achieved. In the first phase, the moving depth was 5 mm, 9 mm or 10 mm, while the feeding speed was set to 100 mm/min and 200 mm/min, respectively. In the second phase, the depths were 10 mm, 15 mm, 19 mm and 20 mm, while the feeding speeds were 900 mm/min, 1200 mm/min, 1800 mm/min and 2000 mm/min, respectively.

The measured force and torque during the fabrication process were constantly monitored and the amount of heat input was calculated as a result of the mechanical energy using Equation (1) according to Amancio-Filho [28]:

(1) $$E_{M}=E_{f}+E_{d}=\int T\omega \mathrm{dt}+\int F\upsilon \mathrm{dt}$$

where *E*_f_ presents the friction energy input of the first phase as a result of torque (*T*) and the angular velocity (ω) and the deformational energy (*E*_d_) of second phase as a result of the axial force (*F*) and the deformation rate (*υ*) and time, respectively. The angular velocity, i.e., the rotational speed was calculated using Equation (2), where *n* presents the rivet rotational speed in revolutions per minute:

(2) $$\omega =\frac{2\pi n}{60}\left[\mathrm{rad}/s\right]$$

After the riveting process, the samples were subjected to optical microscopy observations (Keyence, Osaka, Japan), X-ray examination (Labquip, Wolvey Hinckley, UK) and pull-out tests. The pull-out tests were performed using the universal testing machine Z250 with a maximum load of 250 kN, using TestExpert software, both from Zwick, Ulm, Germany. Afterwards, the samples were cut using the precision saw and prepared for macrostructural observations. From the X-ray images, the dimensions of the fabricated rivets were measured. These values were used to calculate the volumetric ratio-*VR* (0 ≤ *VR* ≤1) using Equation (3) [28,29,31]:

(3) $$VR=\frac{\left(W^{2}-D^{2}\right)D_{p}}{HW^{2}}\left[/\right]$$

**Figure 1:**
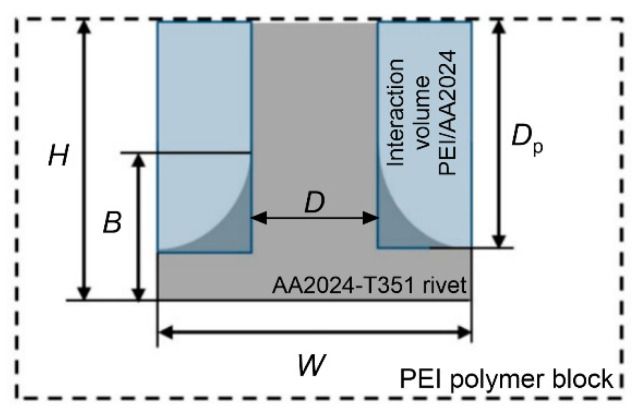
Schematic presentation of volumetric ratio according to Cipriano et al. [28,29].

The volumetric ratio is defined as the ratio between the volume of the plastically deformed rivet and the volume of the polymer offering a mechanical resistance to the pull-out action (Figure 1). Afterwards, the results were analysed and a correlation between the processing parameters, the force and the torque, the volumetric ratio and the anchoring effect was obtained. 

## 3. Results

### 3.1. X-ray Examination and Macroscopic Observations

**Figure 2:**
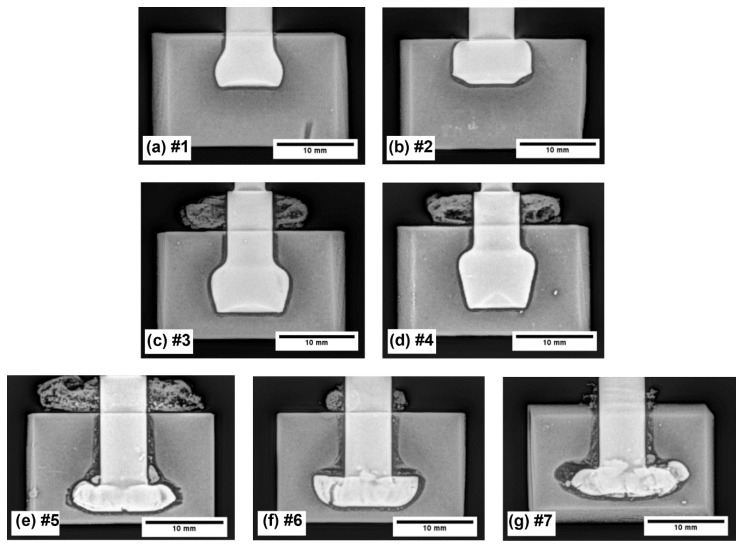
X-ray images of rivet shape and its position inside the base PEI material block (*Note: numbers (#1–#7) by the image captions (**a**–**g**) represent the sample designation using different manufacturing parameters—see Table 1).

Within this study, all of the visually acceptable samples, which were fabricated via the friction riveting process, have been subjected to an X-ray examination, micro-sectional observations and the pull-out tests. Figure 2 depicts the X-ray images of the manufactured samples. As can be clearly observed, the main difference among the sample appearances is the height position of the rivet and its ending shape. 

**Figure 3:**
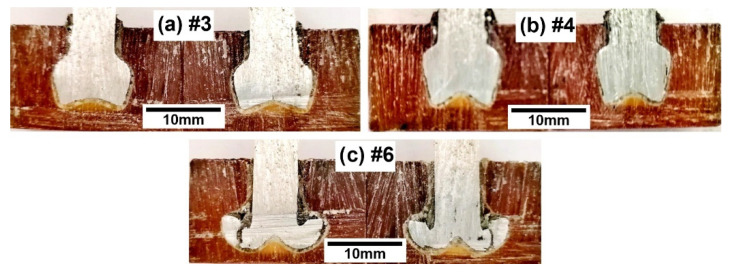
Macrostructural observations of the rivets obtained with different friction riveting parameters; (**a**) parameters No. #3, (**b**) parameters No. #4 and (**c**) parameters No. #6.

The samples that were processed with the friction riveting parameters #1 and #2 (Figure 2a,b) depict bell-shaped rivets, whereas the widest rivet diameter is located toward the bottom of the rivet. Both of the rivets were positioned close to the rivet penetration surface, and they did not reach the desired depths, with there being an insufficient proportion of PEI material above the deformed rivet for sufficient pull-out resistance (Figure 2b). By using the fabrication parameters #3 and #4 (Figure 2c,d), the results that were produced also shows that there were bell-shaped rivets, whereas the widest rivet parameter was not at the bottom. As expected, these two rivets penetrated deeper into the PEI material due to the parameters that were used during their fabrication (Table 1). At the bottom, the centre part of these rivets have a conical shape and missing material could be noted, which was additionally confirmed from an observation of its macro-sections (Figure 3). In contrast, Figure 2e–g depicts the anchoring shape on the bottom of the rivets. All of these rivets have a much wider bottom diameter, and they are located deeper inside of the PEI block. 

More importantly, these samples also depicted the presence of cracks on the rivet shape, due to overly rough parameters being used during the production of the rivets. Cracks or the broken rivet materials, e.g., AA2024-T351, are even more evident in Figure 3c. Based on the macro-sectional observations, it can be noted that within the samples that were fabricated with parameters #3 (Figure 3a) and #4 (Figure 3b), the heat input in the centre of the rivet was lower due to us using smaller relative rotational speeds in combination with a lower friction coefficient. As an outcome, within this region of the PEI sample, the temperature reached lower temperatures and is therefore, stiffer. All of the alloy bar/rivets have consequently formed a conical shape at the bottom. 

In contrast, the sample that was fabricated with parameters #6 (Figure 3c) at a larger distance from the centre of the rivet joint depicts that both the AA2024-T351 and PEI base block material reached higher temperatures during their manufacture. Therefore, the aluminium alloy and PEI were formed more extensively during the riveting process, and the anchoring shape of the rivet was obtained. Despite the presence of the cracks, a higher pull-out force is expected to be needed with such rivet shapes and this will be either confirmed or rejected in the following sections.

### 3.2. Axial Force, Torque and Heat Input

**Figure 4:**
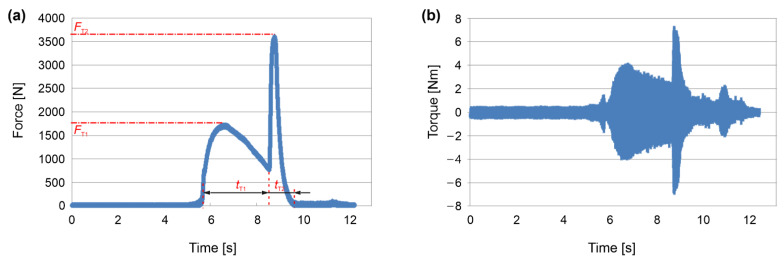
Measured data during friction riveting process using set of parameters #1; (**a**) axial force and (**b**) torque vs. time, respectively.

In each riveting experiment, using a set of specific parameters, the axial force and torque were measured continuously using equipment from Kistler and National Instruments. The typical plots of these measurements are depicted in Figure 4, which shows the friction riveting for parameter #1. A correlation between the axial force and the torque can be seen, with the torque being directly dependent on the axial force and with it being higher due to the increased coefficient of friction. The force and the torque change during the process due to the feeding rate, the coefficient of friction and the temperature-dependent mechanical properties of the two materials that were in contact. The feeding rate, and consequently, the heat input also changed during the process the as friction coefficient was changing. As can be observed from the plot also, the actual durations of the friction riveting process can be determined, which represent the sum of the *t*_T1_ and *t*_T2_ processing time of the first and the second riveting phase, respectively.

**Figure 5:**
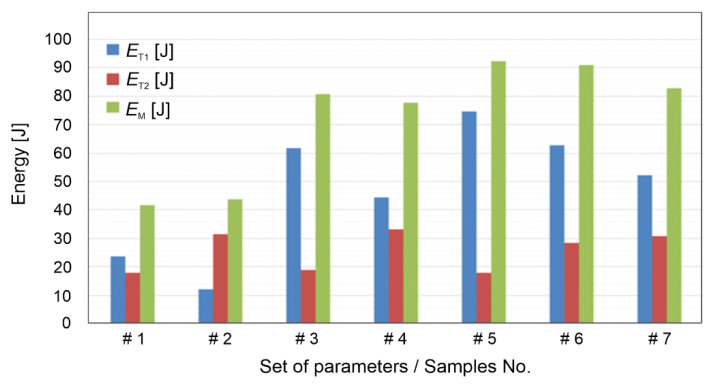
Energy input due to torque (*E*_T1_), angular velocity (*E*_T2_) and combined mech. energy (*E*_M_).

The data of the measured forces and torques were collected, and they are shown in the Table 5. The values from the plots were used to calculate the heat inputs as a result of the mechanical energy according to Equations (1) and (2). These energy inputs for the first and second phases of the friction riveting are shown in Figure 5.

Table 5 and Figure 5 clearly depict that a higher axial force produce a higher torque because of the friction coefficient. Consequently, a higher energy input is generated, which heats the materials that are in contact, thereby causing the sufficient deformation of the rivet. Moreover, by comparing the results of the X-ray images, the processing parameters, the measured axial force and the torque and the calculated energy inputs, the following conclusions can be made: (*i*) In the first phase, a higher axial forces produced a higher torque and consequently, a higher energy input, which heated the rivet and the base material more, and as a result of the feeding rates, it pushed the rivet deeper into the base material. (*ii*) In the second phase, higher penetration depths and lower feeding rates produced a higher deformation of the rivet, which provided an anchoring shape with a wider rivet end. Such a rivet shape is of course desired and beneficial since higher pull-out forces are required to separate the dissimilar riveted joints [22,23,25,28,29,31].

### 3.3. Volumetric Ratio and Pull-Out Force

**Figure 6:**
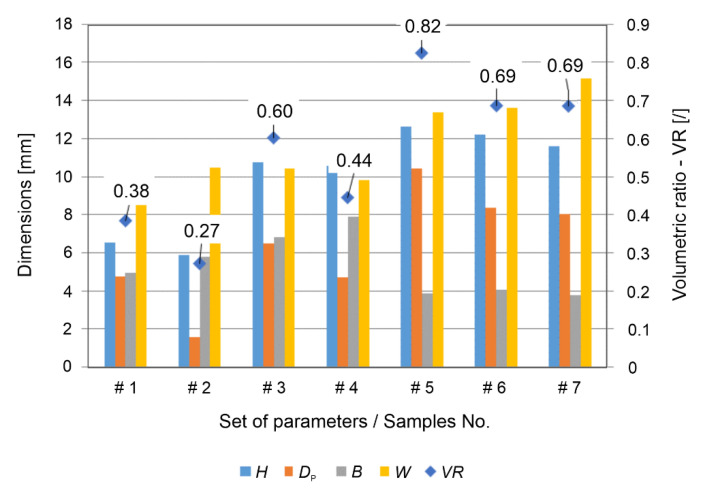
Rivet dimensions obtained from X-ray images according to Figure 1 and volume ratio (*VR*) calculated using Equation (3).

The characteristic dimensions of the rivet shape (*see* Figure 1) were measured on the basis of the X-ray images. These values are depicted in Figure 6 as the rivet penetration depth *H*, the maximum rivet diameter *W*, the depth *D*_p_ and the height of the deformed rivet *B*. From these values, the volumetric ratio according to Equation (3) was determined for all of the manufactured samples. The values that were obtained ranged between 0.27 and 0.82. These values are shown in Figure 6 together with the representative values of the rivet shape dimensions.

**Figure 7:**
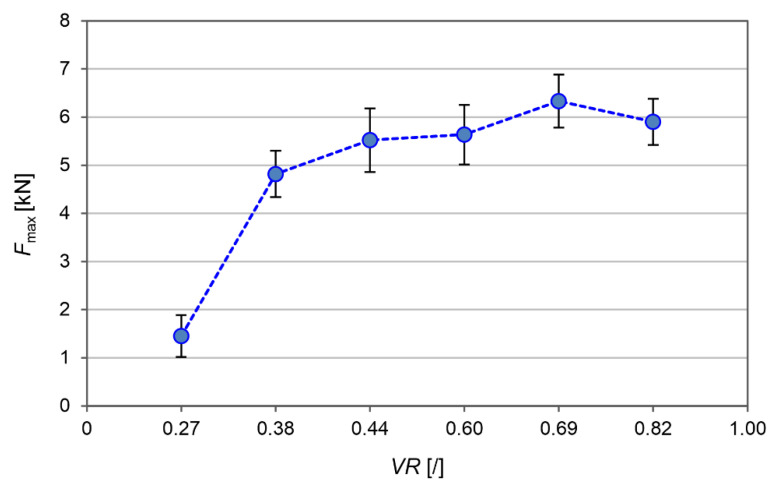
The influence of volume ratio on the rivet pull-out strength.

The obtained joints were subjected to a pull-out test using a universal testing machine (Figure 7). The pull-out forces ranged from 1.5 kN to almost 6.5 kN. The highest joint strength was obtained at a volume ratio of 0.69 with the set of friction riveting parameters #6 and #7. At a higher or lower volume ratio, the pull-out force decreased, thus indicating the “*narrow*” optimum technical window of the process, whereby a difference of only 1 mm in the depth of the movement in the first phase of the friction riveting plays a crucial role.

**Figure 8:**
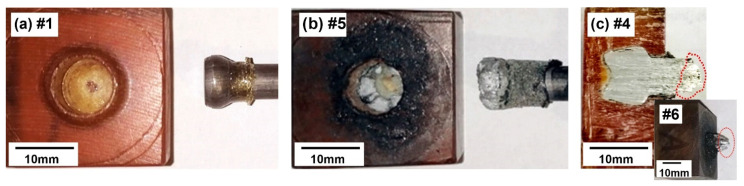
Three main types of joint failures; (**a**) failure in the PEI base material, (**b**) failure in the deformed section of the rivet with an anchor shape and (**c**) failure in the undeformed section of the AA2024-T351 rivet—red colour (*Note: numbers by the image captions represent the sample/parameters designation using different manufacturing parameters—see Table 1).

An inspection of the failure of the joint revealed that there were three different types of failure (Figure 8): (*i*) The pull-out of the rivet due to insufficient penetration depth and geometric properties of the rivet, as in specimens No. #1 and #2 (Figure 8a). In this case, a bell-shaped rivet was positioned on the top of the sample. (*ii*) The breakage of the anchor (deformed) part of the rivet, which was observed in the sample that was processed with parameters No. #5 (Figure 8b), and (*iii*) a failure in the base rivet material’s narrowest part outside of the PEI block (Figure 8c). 

The third type of failure with the breakage of the AA2024-T351 rivet was observed in specimens #3, #4, #6 and #7. In this failure mode, the highest pull-out forces were required to separate the rivet joint, and the highest ultimate pull-out force was observed when the final end shape of the rivet formed an anchor shape, with a volumetric ratio of 0.69 for both of these experiments, i.e., processing parameters #6 and #7. 

## 4. Discussion

The analysis of the processing parameters of the friction riveting process confirmed that with a higher feeding rate in the first phase of the riveting process, the forces and torque are also higher. The torque increases with the increasing the feeding depth of the rivet, as the rivet remains in contact within the PEI block at the given depth for a longer duration. For this reason, more mechanical heat is generated. 

In the second phase of the riveting process, the maximum value of the measured axial processing force reached at a higher feeding rate. The torque increased with the increasing of the feeding speed and the depth of the rivet. The mechanical energies were calculated using data of the measured torque, time and angular velocity. To obtain the rivets with an anchor shape, a higher energy input is required in the first processing phase. 

The visual inspection showed that a different amount of the PEI polymer was pushed to the outside of the PEI block. Since the PEI was semi-transparent, the visual inspection also revealed the provisional rivet shape. An X-ray examination (Figure 2) showed that the rivets were formed at different depths and had two different geometric shapes: (*i*) bell-shaped rivets and (*ii*) anchored rivets (Figure 2 and Figure 3). The bell-shaped rivets indicated good structural integrity with them having no significant cracks on the surface, while the volume ratio was always smaller when it was compared to that of the anchored rivets. The latter also showed a partially cracked surface of the AA2024-T351 rivet within the PEI base block. At the same time, the anchored rivet diameter was always larger than the diameter of the bell-shaped rivet. The calculated values of the volumetric ratio (VR), based on the X-ray analyses, were in the range between 0.38 and 0.82. A higher VR generally means that the rivet has a larger diameter, and consequently, there is more PEI material above the rivet, which results in better anchoring. However, due to the narrow optimal parameters, saturation was observed and unequal rivets with a *VR* that was above 0.69 (parameter set No. #5) required less force to disassemble them during the pull-out test. This can be directly related to the fact that a larger part of the PEI is pushed out of the base polymer block (Figure 2e).

Thus, the highest pull-out force was observed when the final shape of the rivet formed an anchor shape and had the volume ratio of 0.69. In this case, the anchor shape of the rivet with a larger diameter, and at the same time, with there being a reasonable height of the anchor prevented the failure of the anchor itself.

## 5. Conclusions

In this study, the influence of the friction riveting parameters on the formation and strength of dissimilar AA2024-T351/PEI joints was investigated. Based on the results that were obtained, the following conclusions can be drawn:

- The X-ray studies and macroscopic observations have confirmed that it is possible to achieve the desired depth and final shape of AA2024-T351 rivets in the PEI material by controlling the processing parameters.
- The results have confirmed that in the first phase of manufacturing, it is essential to ensure that there is a sufficient axial force and thus, consequently, a sufficient torque for 2.5 to 3 s to achieve the adequate penetration depth of the AA2024-T351 rivet into the PEI base material.
- Proper processing parameters lead to a higher frictional energy input due to the effects of friction and the strong heating of the rivet and the PEI material. Thus, optimal conditions were achieved with the feeding rate of 200 mm/min and a penetration depth of ~10 mm in the first phase of the friction riveting process.
- Furthermore, in the second phase of the process, the deformation energy, which depended on the axial force and the deformation speed, was crucial for the correct formation and anchoring shape of the rivet. For the dissimilar materials that were used in the current study, the optimal anchoring shape of the rivet can be achieved at a penetration depth of ~20 mm and a feed rate of 900 mm/min.
- The analyses of the volume ratio and the pull-out force results confirmed that the highest joint strength was achieved with a maximum pull-out force of up to 6.5 kN at a volume ratio of 0.69. Here, the rivet obtained an anchoring end shape and the failure occurred in the undeformed section of the rivet, which was outside of the PEI block.
- In contrast, the pull-out force decreased at a higher or lower volume ratio, indicating the “*narrow*” optimum technical window of the process.

## Figures and Tables

**Table 1:** Experimental plan for parametric analysis of friction riveting process at constant rivet rotation speed of 19,000 min^−1^.

Parameters/ Sample	Phase 1		Phase 2	
	*z* [mm]	*V*_f_ [mm/min]	*z* [mm]	*V*_f_ [mm/min]
#1	5	100	10	1200
#2	5	200	15	2000
#3	10	200	20	1200
#4	10	200	20	1800
#5	10	200	20	900
#6	9	200	20	900
#7	9	200	19	900

**Table 2:** Chemical composition (in wt.%) of AA2024-T351 [36].

Cu	Mg	Mn	Si	Fe	Zn	Ti	Cr	Al
3.8–4.9	1.2–1.8	0.3–0.9	≤0.5	≤0.5	≤0.25	≤0.15	≤0.1	Bal.

**Table 3:** Mechanical and physical properties of AA 2024-T351 [37].

Property	Value
*R*_m_ [MPa]	425
*R*_p0.2_ [MPa]	310
*E* [GPa]	73.1
Melting temperature [°C]	502–638
Heat treatment temperature [°C]	493
Tempering temperature [°C]	413
Heat conductivity [W/mK]	121

**Table 4:** Mechanical and physical properties of PEI material [25,37].

Property	Value
*R*_m_ [MPa]	1–281
*E* [GPa]	39.4
*μ*_tr_	0.18–0.42
*T*_g_ [°C]	168–220
Heat conductivity [W/mK]	0.036–11
Melting temperature [°C]	171–238
Density [g/cm^3^]	0.05–1.78
Curing temperature [°C]	82.2–150

**Table 5:** Values of force (*F*), torque (*M*) and processing time (*t*) for both phases of friction riveting.

Parameters/ Sample	Rev [min^−1^]	*F*_T1_ [N]	*t*_T1_ [s]	*F*_T2_ [N]	*t*_T2_ [s]	*M*_1_ [Nmm]	*M*_2_ [Nmm]
#1	19,000	1694.16	2.95	3556.63	1.25	4.02	7.22
#2	19,000	3337.65	1.39	10,317.32	0.76	4.36	20.91
#3	19,000	3794.90	3.43	5741.10	0.92	9.06	10.37
#4	19,000	3499.12	2.93	9242,00	0.82	7.62	20.27
#5	19,000	3893.49	2.96	4387.70	1.11	12.65	8.05
#6	19,000	3615.41	2.63	4906.56	1.09	11.96	13.07
#7	19,000	3997.13	2.64	4518.52	1.05	9.92	14.73

*Note: Colour within the table represent the scale: 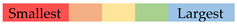

## Data Availability

The data that support the findings of this study are available from the corresponding author upon reasonable request.
